# Prevalence of arrhythmias among children below 15 years of age with congenital heart diseases attending Mulago National Referral Hospital, Uganda

**DOI:** 10.1186/s12872-016-0243-1

**Published:** 2016-04-13

**Authors:** Anthony Batte, Peter Lwabi, Sulaiman Lubega, Sarah Kiguli, Violette Nabatte, Charles Karamagi

**Affiliations:** Child Health and Development Centre, Makerere University College of Health Sciences, P.O Box 6717, Kampala, Uganda; Clinical Epidemiology Unit, Makerere University College of Health Sciences, P.O Box 7072, Kampala, Uganda; Uganda Heart Institute, P.O. Box 7051, Kampala, Uganda; Department of Paediatrics and Child Health, Makerere University College of Health Sciences, P.O Box 7072, Kampala, Uganda

**Keywords:** Arrhythmias, Electrocardiography, Congenital heart diseases

## Abstract

**Background:**

In Uganda, few children with congenital heart diseases (CHD) benefit from early corrective cardiac surgery. These children are at high risk of developing heart failure and electrolyte imbalances; factors which increase their risk of developing arrhythmias. This study aimed to determine the prevalence and factors associated with arrhythmias among children with congenital heart diseases receiving care at Mulago Hospital.

**Methods:**

This was a cross-sectional study carried out from August 2013 to March 2014 at Mulago Hospital. Children were consecutively enrolled into the study. Standard 12-lead electrocardiograms (ECGs) were performed on 194 children with CHD (age range 10 days–15 years). Data was analysed using SPSS 16.0.

**Results:**

Out of 194 children studied, 53/194 (27.3 %, 95 % CI 21.0 – 33.6) children had arrhythmias. Of the CHD children, 44/194 (22.7 %, 95 % CI 16.8 – 28.6) had first degree AV block while 9/194 (4.6 %, 95 % CI 1.7 – 7.6) children had either ectopic atrial rhythm, premature atrial contractions, junctional rhythm, complete atrioventricular (AV) dissociation or premature ventricular contractions. Children using digoxin were more likely to have first degree AV block (OR 3.75, 95 % CI 1.60–8.86) while those aged 5 years and below were less likely to have first degree AV block (OR 0.16, 95 % CI 0.07–0.37).

**Conclusion:**

Arrhythmias are common among children with CHD receiving care from Mulago Hospital. These are associated with digoxin use, child’s age and electrolyte imbalances; factors which can easily be assessed, managed and where possible modified in these children during their care.

## Background

Children with congenital heart diseases (CHD) commonly develop arrhythmias. If they are not identified early and managed appropriately, the arrhythmias contribute to morbidity and mortality among these children [1]. The arrhythmias are a result of either co-existing congenital abnormalities of the specialized conduction system or in response to hemodynamic influences on chamber dimensions, muscle mass and metabolism of myocardial tissue [2].

In low resource countries, very few children with CHD get early corrective heart surgery [3]. Due to delayed surgery, these children develop many complications such as heart failure, severe cyanosis and other long-term sequelae which increase their risk of developing arrhythmias [4]. In addition, children in low resource settings are at an increased risk of malnutrition, HIV and electrolyte imbalances; factors which are known to increase the risk of developing arrhythmias [5–7].

There is currently limited data on the magnitude of arrhythmias among children with congenital heart diseases in low resource countries due to limited access to electrocardiography services in these countries. In Uganda, electrocardiography is not offered as part of the routine care of children with congenital heart diseases because of its limited availability and high cost. We therefore conducted a cross-sectional study to determine the prevalence and factors associated with arrhythmias among children with congenital heart diseases receiving care at Mulago National Referral Hospital, Kampala.

## Methods

### Study site and population

Mulago National Referral Hospital is located in Kampala, the capital city of Uganda. The hospital has the Uganda Heart Institute which employed the only two paediatric cardiologists in the country at the time when the study was carried out. Children with congenital heart diseases from different parts of the country receive care from this hospital.

Children with congenital heart diseases who presented to the hospital between the period of August 2013 and March 2014 were consecutively enrolled into the study. We included all children aged 0–15years with congenital heart disease confirmed on echocardiography by a qualified paediatric cardiologist.

### Sample size calculation

The number of participants was generated using the Kish Leslie (1965) formula for cross-sectional studies [8]. Prevalence of arrhythmias among children with congenital heart disease in other studies ranges from 6.3 % (before surgery) to 12 % (in the late post-operative follow up period) [9]. The sample size required, n, was calculated using the formula *n* = pqz^2^/d^2^, where p is the prevalence of arrhythmias among children with congenital heart defects (*p* = 0.12); q = 1-p; z is 1.96 (for 5 % alpha error); and d is precision which is 0.05 (permissible margin of error at 5 % level of statistical significance). This gave a total of 162 children with congenital heart defects. We incorporated a non-response rate/missing data of 20 % [10] and this gave a total required study population of 194 children.

### Data collection

Standardized pretested and pre – coded interviewer – administered data collection tools were used in data collection. Data collection was done by the principal investigator (AB) and a research assistant who was a medical officer with two years of experience in management of children with heart diseases.

### Clinical history

Information collected included child’s social demographic characteristics and information concerning the medication the child was using in the period one week prior to the interview (heart failure medication). All children who were enrolled into the study were assessed for features of heart failure by the principal investigator or research assistant. The severity of heart failure was graded using the modified Ross score. Patients with a score of 0 to 6 were classified as no or mild heart failure while those with a score of above 6 were classified as having moderate or severe heart failure [11, 12].

### Laboratory investigations

A complete blood count was done for each child using a Celltac E MEK-7222 automatic hematology analyzer. The World Health Organization age specific values for hemoglobin levels were used to classify children as having mild, moderate or severe anaemia [13]. We analysed serum electrolytes using automated clinical chemistry analyzer (Cobas® Integra 400 plus model Roche, Germany). Interpretation of serum electrolyte values was made with reference to age specific reference values described by Pesce 2007 [14].

We tested all children for HIV using standard rapid testing kits. The procedure involved an initial screen Alere™ HIV-1/2 Determine® followed by STAT-PAK® HIV-1/2; the third rapid test Uni-Gold™ Recombigen® HIV was used as a tie breaker. For children under 18 months with a positive HIV serology test and of unknown HIV status (children without an HIV DNA PCR report done in the past 3 months), blood was sent for DNA PCR to confirm presence or absence of HIV.

### Electrocardiography procedure

The electrocardiography was conducted using ECG machines (Mortara ELI 350 model, USA and MCA 1200 model, USA). Standard 12-lead electrocardiography was performed in supine position (at 25 mm/s. and voltage of 10 mm/mV). ECGs were carried out by a technician who is a professional nurse working with the Uganda Heart Institute; she was trained in the study methodologies before the start of the study. The electrocardiograms were interpreted by two paediatric cardiologists (PL and SL). Each electrocardiograph was read and interpreted by any one of the cardiologists who was available on duty on a given day during the study period; and the cardiologist’s interpretation was taken as the final interpretation for a given ECG.

### Statistics

Data was entered using Epi data version 2.1b and then exported to SPSS 16.0 for analysis. Descriptive statistics were generated as frequencies and distributions. Associations were derived between the independent and the outcome variables (arrhythmias). At bivariate analysis, factors with a p value less than 0.2 were entered into the final logistic regression model using a model of 7 events per variable [15] and analysed using the backward likelihood ratio approach. Variables with p values less than 0.05 were considered significant.

## Results

### Baseline characteristics

A total of 194 children aged 10 days to 15 years were recruited (median age 2.4 years, IQR 1- 5.3). Only 2 children (1 %) were HIV positive and none of them was on antiretroviral therapy. Majority of the children 111 (57.2 %) were female. A large number of the children presented with ventricular septal defects 48/194 (24.7 %), patent ductus arteriosus 38/194 (19.6 %) and tetralogy of Fallot 25/194 (12.9 %) as shown in Table 1. In total, these three diagnoses constituted 57.2 % of the congenital heart diseases among the children. Majority of the children had a single heart anomaly 163/194 (84 %) while fewer children 31/194 (16 %) had multiple structural heart anomalies as shown in Tables 1 and 2.

**Table 1:** Children with single structural congenital heart anomalies

Abnormality	Frequency	Percentage^a^
Ventricular Septal Defect (VSD)	48	24.7
Patent Ductus Arteriosus (PDA)	38	19.6
Tetralogy of Fallot	25	12.9
Complete atrio-ventricular defect	14	7.2
Atrial septal defect (ASD)	10	5.2
Double outlet right ventricle	9	4.6
Truncus arteriosus	8	4.1
Pulmonary stenosis	4	2.1
Ligated arteriosus	3	1.5
Aortic stenosis	2	1.0
Mitral valve prolapse	1	0.5
Aorto-pulmonary window	1	0.5
Total	163	84.0

^a^Percentage is based on the total number of children in the study (194)

**Table 2:** Children with multiple structural congenital heart anomalies

Abnormality	Frequency	Percentage^a^
Ventricular septal defect + atrial septal defect + patent ductus arteriosus	7	3.6
Pulmonary atresia + ventricular septal defect	6	3.1
Ventricular septal defect + patent ductus arteriosus	4	2.1
Tricuspid atresia + ventricular septal defect + atrial septal defect	4	2.1
Atrial septal defect + patent ductus arteriosus	3	1.5
Ventricular septal defect + atrial septal defect	2	1.0
Common atrium + patent ductus arteriosus	1	0.5
Aorto-right ventricular fistula + patent ductus arteriosus	1	0.5
Double inlet left ventricle + Transposition of the Great Arteries	1	0.5
Dextrocardia + Common atria + ventricular septal defect	1	0.5
Ventricular septal defect + atrial septal defect closed by patch	1	0.5
Total	31	16.0

^a^Percentage is based on the total number of children in the study (194)

### Arrhythmias among the children with congenital heart disease

Arrhythmias were found in 53/194 (27.3 %, 95 % CI 21.0 – 33.6) children as shown in Table 3. Of these children, 44/194 (22.7 %, 95 % CI 16.8 – 28.6) had first degree AV block while 9/194 (4.6 %, 95 % CI 1.7 – 7.6) had either ectopic atrial rhythm, premature atrial contractions, junctional rhythm, complete atrioventricular (AV) dissociation or premature ventricular contractions; the clinical and laboratory characteristics of these children are shown in Table 4.

**Table 3:** Arrhythimias found among the 194 children with congenital heart diseases

Arrhythmia	Frequency	Percentage^a^ (95 % CI)
First degree A-V block	44	22.7 (16.8 – 28.6)
Ectopic A-V rhythm	3	1.5 (0.2 – 3.2)
Premature atrial contractions	3	1.5 (0.2 – 3.2)
Junctional rhythm	1	0.5 (0.5 – 1.5)
Complete A-V block	1	0.5 (0.5 – 1.5)
Premature ventricular contractions	1	0.5 (0.5 – 1.5)
Total	53	27.3 (21.0 – 33.6)

*CI* confidence interval, *A*-*V* atrio-ventricular

^a^Percentage is based on the total number of children in the study (194)

**Table 4:** Characteristics of children with arrhythmias other than first degree AV block

Child’s age	Sex	Arrhythmia	Heart defect	Serum Electrolytes (mmol/L)	Heart failure	Anaemia
2 years	F	Ectopic atrial rhythm	Truncus arteriosus	Normal	Severe	Mild
3 years 8 months	M	Ectopic atrial rhythm	Pulmonary stenosis	↑ Mg^2+^ (1.17) ↓ K^+^ (2.30) ↓ Ca^2+^ (1.5)	No	Moderate
4 months	F	Ectopic atrial rhythm	TOF	↓ Na^+^ (131.3)	No	Moderate
3 years	M	PACs	ASD	↑ Na^+^ (147.8)	No	No
3 months	F	PACs	VSD	↑ Mg^2+^ (1.10) ↓ Na^+^ (131.0)	Severe	Moderate
10 months	M	PACs	Common atrium + PDA	Normal	Severe	No
6 years	F	Junctional rhythm	Dextrocardia + common atrium + VSD	↑ Mg^2+^ (1.0)	Severe	No
2 years 1 months	M	Complete AV block	TOF	Normal	Moderate	No
14 years	M	PVCs	Aortic stenosis	↓ Ca^2+^ (1.8) ↓ Na^+^ (127.0)	Severe	Severe

*Abbreviations*: *F* female, *M* male, *PACs* premature atrial contractions, *PVCs* premature ventricular contractions, *AV* atrial ventricular. ↓ - Lower than the reference value for age, ↑ - Higher than the reference value for age. Values of calcium are total serum calcium levels

Of the children who had first degree AV block, all had never had corrective heart surgery, none of them had hypercalcemia and all were HIV negative. At bivariate analysis, factors that were associated with first degree AV block included age 5 years and below, use of digoxin and hypokalemia as shown in Tables 5 and 6. At adjusted analysis, factors that were associated with first degree AV block were digoxin use (OR 3.75, 95 % CI 1.60–8.86) and age 5 years and below (OR 0.16, 95 % CI 0.07–0.37) as shown in Table 7.

**Table 5:** Association between clinical-demographic variables and first degree A-V block in children with congenital heart disease

Variable	First Degree A-V Block		Unadjusted OR (95 % CI)	*p*-value
	Yes, n (%)	No, n (%)		
Age				
≤5 years	22 (15.3 %)	122 (84.7 %)	0.21 (0.10–0.44)	<0.001
> 5 years	22 (46.8 %)	25 (53.2 %)		
Type of heart disease				
Cyanotic	11 (20.4 %)	43 (79.6 %)	0.81 (0.37–1.74)	0.583
Acyanotic	33 (24.1 %)	104 (75.9 %)		
Heart failure				
Mod/severe	20 (26.3 %)	56 (73.7 %)	1.35 (0.69–2.67)	0.382
No/mild	24 (20.9 %)	91 (79.1 %)		
Digoxin use				
Yes	17 (34 %)	33 (66 %)	2.18 (1.06–4.47)	0.034
No	27 (19.2 %)	114 (80.8 %)

**Table 6:** Association between serum electrolytes and first degree A-V block in children with congenital heart disease

Variable	First Degree A-V Block		Unadjusted OR (95 % CI)	*p*-value
	Yes, n %)	No, n (%)		
Hyperkalemia				
Yes	2 (9.5 %)	19 (90.5 %)	0.32 (0.07–1.44)	0.138
No	40 (24.7 %)	122 (75.3 %)		
Hypokalemia				
Yes	4 (57.1 %)	3 (42.9 %)	4.84 (1.04–22.57)	0.045
No	38 (21.6 %)	138 (78.4 %)		
Hypernatremia				
Yes	13 (32.5 %)	27 (67.5 %)	1.88 (0.87–4.07)	0.110
No	30 (20.4 %)	117 (79.6 %)		
Hyponatremia				
Yes	9 (16.4 %)	46 (83.6 %)	0.56 (0.25–1.27)	0.168
No	34 (25.8 %)	98 (74.2 %)		
Hypermagnesemia				
Yes	9 (21.4 %)	33 (78.6 %)	0.84 (0.36–1.95)	0.678
No	30 (24.6 %)	92 (75.4 %)

**Table 7:** Logistic Regression for factors associated with first degree A-V block in children with congenital heart disease

Variable		Adjusted OR (95 % CI)	*p*-value
Age	≤ 5 years	0.16 (0.07–0.37)	<0.001
	> 5 years		
Digoxin	Yes	3.75 (1.60–8.86)	0.003
	No		
Hyperkalemia	Yes	0.37 (0.08–1.75)	0.210
	No		
Hypokalemia	Yes	3.35 (0.59–18.87)	0.171
	No		
Hypernatremia	Yes	1.15 (0.44–3.04)	0.773
	No		
Hyponatremia	Yes	0.75 (0.29–1.92)	0.545
	No		

## Discussion

In this study, arrhythmias were found in 27.3 % of children; with 22.7 % having first degree AV block while 4.6 % had either ectopic atrial rhythm, premature atrial contractions, junctional rhythm, complete atrioventricular (AV) dissociation or premature ventricular contractions (PVCs). This prevalence of arrhythmias with the exception of first degree AV block is comparable to a study by Ringel and colleagues 1984 who detected arrhythmias in 4/64 (6.3 %) children (age one week to 16 years) with congenital heart disease attending University of Merryland hospital [9]. However, this prevalence is lower than the prevalence of arrhythmias detected by holter ECGs, which show a prevalence of up to 41.2 % in infants and children with congenital heart diseases before surgical correction of the heart defects [16]. Holter ECGs are not readily available in Uganda and other developing countries and thus the use of standard 12 lead ECG is employed.

Complete AV dissociation, a life threatening arrhythmia was detected in one child (0.5 %). This arrhythmia in association with structural heart disease has a case fatality rate of 29 % in infancy and 10 % in childhood [17]. Complete AV dissociation has been described in children with L-TGA and in children with CAVC [18, 19]. However, in our study this arrhythmia was found in a child with TOF. There is limited literature on occurrence of complete AV dissociation in children with TOF although studies have describe this arrhythmia among fetuses with TOF [20, 21]. This child with complete AV dissociation had normal serum electrolytes (sodium, potassium, magnesium and total serum calcium) implying that imbalances of these serum electrolytes had no role in predisposing the child to complete AV dissociation.

In our study, prevalence of ectopic atrial rhythm was 1.5 %, PACs 1.5 %, junctional rhythm 0.5 % and that of PVCs was 0.5 %. Similar studies among children with congenital heart diseases detected comparable prevalence of these arrhythmias. Prevalence of PVCs being at 1.5 % among children with congenital heart diseases [9]. Ectopic atrial rhythm has been reported in children with TOF at a prevalence of 1/15 (6.7 %) [22]. PVCs have been described in children with VSD and CAVC while PACs and junctional rhythm have been reported in individuals with TGA [19, 23].

The three children with PACs in our study had either normal or mild electrolyte imbalances (one had mild hypernatraemia for age while the second child mild hypernatraemia and mild hypermagnesaemia) which may not have predisposed these children to having PACs.

Significant electrolyte imbalances were seen in a child with ectopic atrial rhythm (hypermagnesaemia, severe hypokalemia and severe hypocalcemia) and the child with PVCs (severe hyponatraemia and severe hypocalcemia). Hypermagnesemia is rare in children and it is often iatrogenic following excessive intake of magnesium. There is limited data on association of hypermagnesimia with ectopic atrial rhythms although some reports have associated hypermagnesemia with first degree AV block [24]. Arrhythmias associated with hypokalemia include a prolonged QT interval, ventricular extrasystoles, and malignant ventricular arrhythmias such as ventricular tachycardia, torsades de pointes and ventricular fibrillation [25]. There are no previous reports associating ectopic atrial rhythm with hypokalemia. However, in patients with underlying heart disease, even mild-to-moderate hypokalemia increases their likelihood of cardiac arrhythmias [26], and this could therefore have predisposed this child to development of an atrial rhythm. Hypocalcaemia causes prolongation of QTc interval and this is directly proportional to the degree of hypocalcaemia [27], there are no reports which associate hypocalcemia with ectopic atrial rhythm or PVCs. This may thus mean that hypocalceamia may not have had any role in predisposing these children to ectopic atrial rhythm and PVCs. The child with PVCs had severe severe hyponatraemia in addition to severe hypocalcemia. Patients with hyponatremia are more prone to cardiac arrhythmias [28] and thus this might have increased the risk of this child developing PVCs.

In our study, prevalence of first degree AV block was 22.7 %. A higher prevalence of first degree AV block (44.4 %) among children and young adults with congenital heart diseases was described by Waldo and colleagues, 1974 in New York [29]. Waldo and colleagues studied 30 children and young adults of ages 2 years to 25 years with various congenital heart diseases. The higher prevalence of first degree AV block in their study compared to the prevalence we have found in our study could be due to the difference in the age groups since Waldo and colleagues included individuals above age 15 years and also because they had a smaller sample size.

There is generally paucity of data on the prevalence of first degree AV block among children with congenital heart diseases. This is because in the past, first degree AV block was considered a benign condition. However, recently first degree AV block has been reported to be associated with increased risk of atrial fibrillation, pacemaker implantation and all-cause mortality especially in adults [30]. Patients with a borderline first-degree AV block during normal sinus rhythm may also have dual AV node physiology and may be at higher risk for clinical atrioventricular nodal re-entry tachycardia (AVNRT) [31]. In Uganda many children with congenital heart diseases grow into adulthood without corrective surgery. Our study does not provide information on how first degree AV block affects these children as they grow with congenital heart diseases. However, these results mean that electrocardiography should be done regularly for these children and there is need for further research to assess the impact of first degree AV block on these children as they grow into adulthood.

In this study, use of digoxin and age were independently associated with first degree AV block. One of the possible explanations for the association between digoxin use and first degree AV block in this study could be that first degree AV block was a manifestation of digoxin toxicity in these children. In this study, we did not measure serum digoxin levels for children who had first degree AV block, which was a limitation. However, digoxin toxicity is known to cause first degree AV block [32].

In this study, children aged 5 years and below were less likely to have first degree AV block compared to children above 5 years. This could be because young children are less likely to have digoxin toxicity. The younger the child, the higher the serum levels of digoxin which can be tolerated before manifestations of digoxin toxicity occurs [32]. The other reason why older children have a higher prevalence of first degree AV block may also be because these children have lived longer with un-operated congenital heart disease and are more likely to present with arrhythmias as a complication of the congenital heart defects.

This study was carried out among children in Mulago Hospital; a setting where children with congenital heart diseases rarely have ECGs done during their care. These results therefore provide an understanding of the prevalence of arrhythmias among the children who are managed in the hospital. The study also shows some of the factors associated with these arrhythmias such as age of the child, digoxin use and electrolyte imbalances. These are factors that can easily be assessed among the children who receive care at Mulago Hospital and in other similar resource limited settings.

### Study limitations

This study was carried out using a standard 12 lead ECG and not a holter ECG thus the prevalence of arrhythmias reported may be lower than the actual prevalence which would be detected using holter ECGs.

In this study we did not determine serum digoxin levels to assess for digoxin toxicity. This limited our ability to determine whether the prevalence of first degree AV block was a feature of digoxin toxicity.

This study was unable to assess other factors which could predispose these children to arrhythmias such as genetic factors, infections and in cases of neonates factors such as congenital infections, maternal diabetes and prematurity.

## Conclusion

Arrhythmias are relatively common among children with congenital heart diseases presenting at Mulago hospital; occurring in approximately one in four children with congenital heart diseases. Factors associated with these arrhythmias include age of the child, digoxin use and electrolyte imbalances. This study therefore provides an understanding of the need to routinely assess children with congenital heart disease for arrhythmias and the various factors associated with these arrhythmias as the children receive routine care at the hospital. Future research can be done using a holter ECG to provide a more comprehensive analysis of the arrhythmias among children with congenital heart diseases in similar resource limited settings.

### Ethical approval and consent

This study was approved by the Makerere University School of Medicine Research and Ethics Committee (SOMREC) and the Uganda National Council for Science and Technology. All caretakers of children provided informed consent for the children before recruitment into the study. In addition, children aged 8 years and above provided assent before participating in the study.

### Availability of data and materials

All relevant data supporting the conclusions of this article is included within the article.

## Abbreviations

ASD: atrial septal defect
AV: atrial ventricular
AVNRT: atrioventricular nodal re-entry
CAVC: common atrial- ventricular canal defect
CHD: congenital heart disease
ECG: electrocardiography
HIV: human immune-deficiency virus
L-TGA: L transposition of great arteries
PACs: premature atrial contractions
PVCs: premature ventricular contractions
TOF: Tetralogy of Fallot
VSD: ventricular septal defect
VT: ventricular tachycardia

## References

[1] Liberthson RR (1996). Sudden death from cardiac causes in children and young adults. N Engl J Med.

[2] Szili-Torok T, Kornyei L, Jordaens LJ (2008). Transcatheter ablation of arrhythmias associated with congenital heart disease. J Interv Card Electrophysiol.

[3] Yacoub MH (2007). Establishing pediatric cardiovascular services in the developing world. Circulation.

[4] Mocumbi AO, Lameira E, Yaksh A, Paul L, Ferreira MB, Sidi D (2011). Challenges on the management of congenital heart disease in developing countries. Int J Cardiol.

[5] Olivares JL, Vázquez M, Rodríguez G, Samper P, Fleta J (2005). Electrocardiographic and echocardiographic findings in malnourished children. J Am Coll Nutr.

[6] Dei Cas L, Metra M, Leier CV (1995). Electrolyte disturbances in chronic heart failure: metabolic and clinical aspects. Clin Cardiol.

[7] Lubega S, Zirembuzi G, Lwabi P (2007). Heart disease among children with HIV/AIDS attending the paediatric infectious disease clinic at Mulago Hospital. Afr Health Sci.

[8] Kish L (1965). Survey Sampling.

[9] Ringel RE, Kennedy HL, Brenner JI, Roberts GS, Berman MA (1984). Detection of cardiac dysrhythmias by continuous electrocardiographic recording in children undergoing cardiac surgery. J Electrocardiol.

[10] Naing L, Winn T, Rusli B (2006). Practical issues in calculating the sample size for prevalence studies. Arch Orofac Sci.

[11] Ross RD, Bollinger RO, Pinsky WW (1992). Grading the severity of congestive heart failure in infants. Pediatr Cardiol.

[12] Reithmann C, Reber D, Kozlik-Feldmann R, Netz H, Pilz G, Welz A, Werdan K. A post-receptor defect of adenylyl cyclase in severely failing myocardium from children with congenital heart disease. Eur J Pharmacol. 1997;330(1):79–86. 10.1016/s0014-2999(97)10131-5 9228416

[13] World Health Organization: Haemoglobin concentrations for the diagnosis of anaemia and assessment of severity. Vitamin and Mineral Nutrition Information System*.* Geneva 2011.

[14] Pesce M. Reference ranges for laboratory tests and procedures. In Stanton B, Berhman K, Stanton J (Eds). Nelson textbook of Paediatrics. 18th Edition, Chapter 715. Elsevier 2007.

[15] Vittinghoff E, McCulloch CE (2007). Relaxing the rule of ten events per variable in logistic and Cox regression. Am J Epidemiol.

[16] Grosse-Wortmann L, Kreitz S, Grabitz RG, Vazquez-Jimenez JF, Messmer BJ, von Bernuth G, Seghaye M-C. Prevalence of and risk factors for perioperative arrhythmias in neonates and children after cardiopulmonary bypass: continuous holter monitoring before and for three days after surgery. J Cardiothorac Surg. 2010;5(1):85. 10.1186/1749-8090-5-85 PMC2974677 20955589

[17] Michaëlsson M, Riesenfeld T, Jonzon A (1997). Natural history of congenital complete atrioventricular block. Pacing Clin Electrophysiol.

[18] Walsh EP (2007). Interventional electrophysiology in patients with congenital heart disease. Circulation.

[19] Khairy P, Balaji S (2009). Cardiac arrhythmias in congenital heart diseases. Indian pacing and electrophysiology journal.

[20] Stewart P, Tonge H, Wladimiroff J (1983). Arrhythmia and structural abnormalities of the fetal heart. Br Heart J.

[21] Jaeggi E, Hornberger L, Smallhorn J, Fouron JC (2005). Prenatal diagnosis of complete atrioventricular block associated with structural heart disease: combined experience of two tertiary care centers and review of the literature. Ultrasound Obstet Gynecol.

[22] Paul O, MYERS GS, CAMPBELL JA (1951). The electrocardiogram in congenital heart disease a preliminary report. Circulation.

[23] Khairy P, Marelli AJ (2007). Clinical use of electrocardiography in adults with congenital heart disease. Circulation.

[24] Harker HE, Majcher TA (2000). Hypermagnesemia in a pediatric patient. Anesth Analg.

[25] Slovis C, Jenkins R, Morris F, Edhouse J, Brady WJ, Camm J (2003). Conditions not primarily affecting the heart. Abc of Clinical Electrocardiography.

[26] Gennari FJ (1998). Hypokalemia. N Engl J Med.

[27] RuDusky BM (2001). ECG abnormalities associated with hypocalcemia. CHEST Journal.

[28] Epstein FH, Schrier RW, Abraham WT (1999). Hormones and hemodynamics in heart failure. N Engl J Med.

[29] Waldo AL, Kaiser GA, Bowman FO, Malm JR (1973). Etiology of prolongation of the PR interval in patients with an endocardial cushion defect further observations on internodal conduction and the polarity of the retrograde P wave. Circulation.

[30] Cheng S, Keyes MJ, Larson MG, McCabe EL, Newton-Cheh C, Levy D, Benjamin EJ, Vasan RS, Wang TJ. Long-term outcomes in individuals with prolonged PR interval or first-degree atrioventricular block. Jama. 2009;301(24):2571–7. 10.1001/jama.2009.888 PMC2765917 19549974

[31] Schlechte EA, Boramanand N, Funk M (2008). Supraventricular tachycardia in the pediatric primary care setting: Age-related presentation, diagnosis, and management. J Pediatr Health Care.

[32] Hastreiter AR, van der Horst RL, Chow-Tung E (1984). Digitalis toxicity in infants and children. Pediatr Cardiol.

